# Membrane capacitance recordings resolve dynamics and complexity of receptor-mediated endocytosis in Wnt signalling

**DOI:** 10.1038/s41598-019-49082-4

**Published:** 2019-09-10

**Authors:** Vera Bandmann, Ann Schirin Mirsanaye, Johanna Schäfer, Gerhard Thiel, Thomas Holstein, Melanie Mikosch-Wersching

**Affiliations:** 1 Department of Biology, Technische Universität Darmstadt, Schnittspahnstrasse 3, 64287 Darmstadt, Germany; 2 Centre for Organismal Studies, University of Heidelberg, Im Neuenheimer Feld 230, Heidelberg, 69120 Germany

**Keywords:** Endocytosis, Morphogen signalling

## Abstract

Receptor-mediated endocytosis is an essential process in signalling pathways for activation of intracellular signalling cascades. One example is the Wnt signalling pathway that seems to depend on endocytosis of the ligand-receptor complex for initiation of Wnt signal transduction. To date, the roles of different endocytic pathways in Wnt signalling, molecular players and the kinetics of the process remain unclear. Here, we monitored endocytosis in Wnt3a and Wnt5a-mediated signalling with membrane capacitance recordings of HEK293 cells. Our measurements revealed a swift and substantial increase in the number of endocytic vesicles. Extracellular Wnt ligands specifically triggered endocytotic activity, which started immediately upon ligand binding and ceased within a period of ten minutes. By using specific inhibitors, we were able to separate Wnt-induced endocytosis into two independent pathways. We demonstrate that canonical Wnt3a is taken up mainly by clathrin-independent endocytosis whereas noncanonical Wnt5a is exclusively regulated via clathrin-mediated endocytosis. Our findings show that membrane capacitance recordings allow the resolution of complex cellular processes in plasma membrane signalling pathways in great detail.

## Introduction

Wnt signalling is a highly-conserved signalling pathway with important functions in development, tissue-homeostasis, stem cell biology and many diseases, including cancer. After three decades of dedicated research we have come to understand many of the fundamental components of Wnt signalling pathways. However, it remains puzzling how so many different processes may be regulated by only one system.

Wnt signalling in principle is highly complex because it is mediated by the interplay of a wide variety of ligands, receptors, co-receptors, antagonists, agonists and intracellular factors, which are deeply embedded in metazoan genomes^1–4^ and interact in a distinct manner. The secreted Wnt proteins activate different downstream pathways, which are traditionally classified as canonical (ß-catenin dependent) and noncanonical (ß-catenin independent). In the canonical Wnt pathway, binding of the extracellular Wnt ligand to the Frizzled receptor leads to the formation of a complex with the co-receptor Lrp5/6. This complex recruits the scaffolding proteins Dishevelled and Axin, as well as GSK3 and several other intracellular components, to build up the Lrp6-signalosome. Upon formation, the Lrp6-signalosome inhibits the phosphorylation of ß-catenin, thus marking it for proteasomal degradation. As a result, there is stabilization of ß-catenin in the cytosol, which regulates various Wnt target genes upon translocation into the nucleus^5^. In contrast, the noncanonical Wnt/PCP pathway utilises Frizzled receptors to activate Dishevelled and regulates various downstream effectors such as small GTPase’s of the Rho and Rac subfamilies, the CaMKII and the PKC pathway^6^.

Cellular mechanisms add another layer of complexity to Wnt signalling, for instance endocytosis of the activated receptors^7,8^. Recent reports show that Wnt signalling can be inhibited when endocytosis of the ligand-receptor complex is blocked^9–16^. Thus, endocytosis is not only necessary for the degradation of ligand-receptor complexes but also crucial for signal activation. The underlying mechanism for this is, however, unclear. There may be several explanations, e.g. the ligand-receptor complex might require acidification in the endocytic vesicles for activation or internalisation is required for the interaction with cofactors. Nevertheless, the mode of uptake of Wnt ligands bound to their receptor is still unknown.

Despite this gap in basic knowledge, a wealth of data has demonstrated distinct differences in the endocytosis of canonical and noncanonical Wnt signalling. Both clathrin-mediated endocytosis and caveolin-dependent endocytosis are involved in canonical Wnt signalling^17^. After formation of Lrp6-signalosomes in response to Wnt3a, these are internalised through a caveolin-mediated route^12,18–20^ but Wnt3a has also been shown to trigger clathrin-mediated endocytosis^9,11,21,22^. Endocytosis of the canonical Wnt8 induced Lrp6-signalosome *via* clathrin-mediated endocytosis has been discovered in zebra fish^15^. For the noncanonical Wnt5a, only clathrin-mediated endocytosis has been reported^23–27^. Ohkawara *et al*.^28^ demonstrated that clathrin-dependent endocytosis of Wnt5a is essential for Wnt/PCP-signalling in *Xenopus*.

Knowledge of receptor-mediated endocytosis following Wnt stimulation is so far restricted to insights from microscopic and/or biochemical analyses. However, these approaches are indirect and limited to the investigation of the endpoint of endocytosis. They lack information on the primary process of endocytosis and fail to provide insights into the dynamics and the temporal resolution of the endocytic process. However, endocytosis may be monitored with high spatial and temporal resolution with recordings of the membrane capacitance^29,30^. In fact, the level of detail provided by membrane capacitance measurements is sufficient to analyse individual vesicle fission events on the plasma membrane in living cells. The method takes advantage of the fact that exo- and endocytosis are associated with changes in plasma membrane area, which, in turn, generate proportional changes in the electrical membrane capacitance (Cm). Thus, even the fission and fusion of single endo- and exocytic vesicles can be resolved in real time in single living cells^31^. Here, we employed the membrane capacitance technique to resolve Wnt receptor-mediated endocytosis with high temporal and spatial resolution in real time. We were able to demonstrate that challenging of HEK293 cells with a canonical (Wnt3a) and a noncanonical ligand (Wnt5a) triggers an immediate increase in endocytosis of small vesicles and that both ligands use separate endocytic pathways; while Wnt5a is endocytosed by clathrin-coated vesicles, Wnt3a takes a clathrin-independent endocytic pathway. Thus, our data provide the first direct measurements of endocytotic processes in Wnt signalling on the cellular level.

## Results

### Characterisation of receptor-mediated endocytosis of Wnt3a and Wnt5a in whole cell mode in HEK293 cells

**Figure 1:**
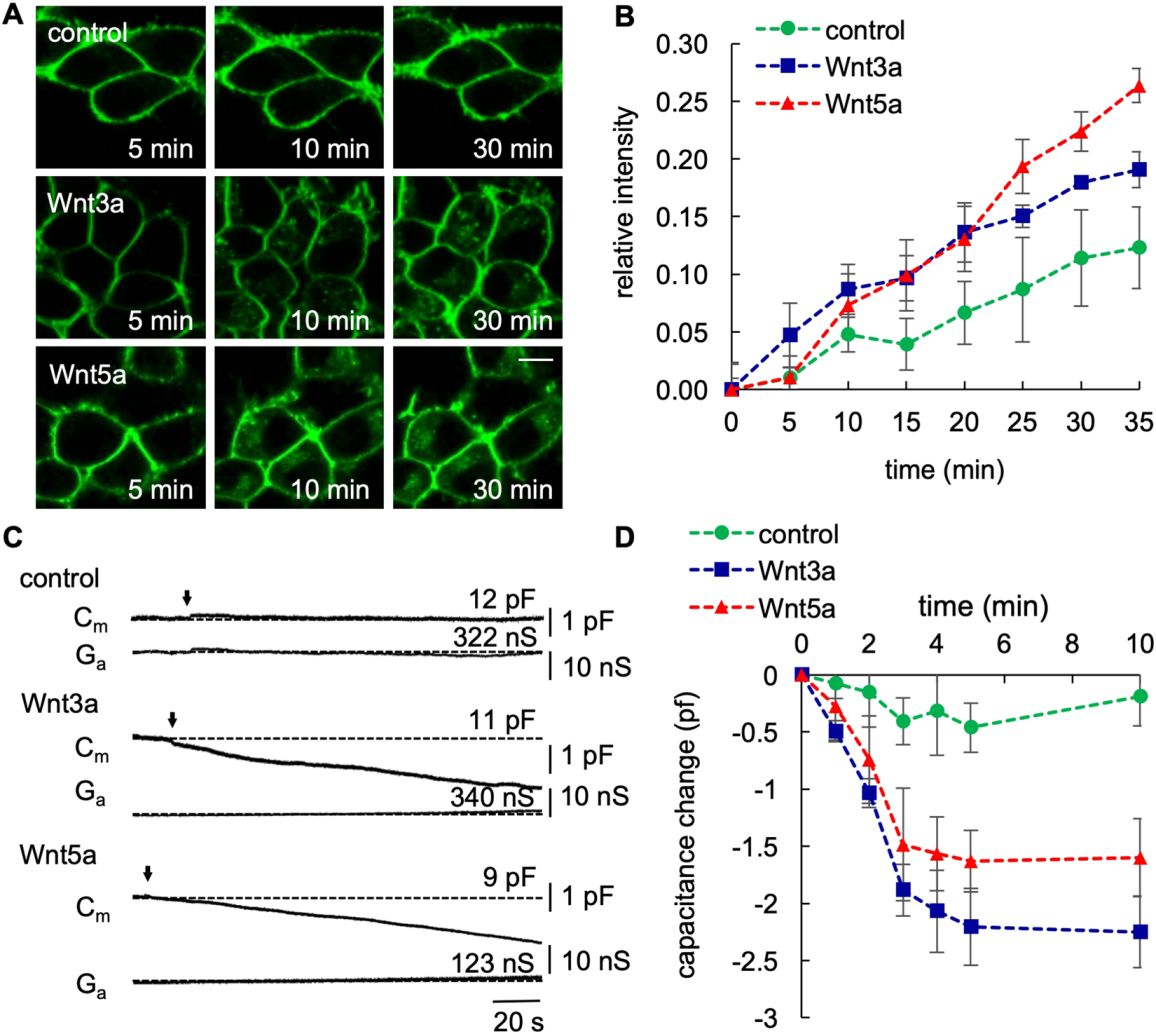
(**A**) Fluorescent image of FM 4-64 endocytosis in control or Wnt-treated HEK293 cells. Cells were incubated for 1 minute with 10 µM of the FM 4-64 dye before addition of 5 ng/ml of the Wnt3a or Wnt5a ligands. Scale bar = 10 µm. (**B**) Quantification of the effect of canonical Wnt3a and noncanonical Wnt5a on the uptake of FM4-64. The relative fluorescent intensity is given as the ratio of intracellular fluorescence to whole-cell fluorescence. In control cells, the relative fluorescent intensity increased over time and shows the steady state uptake of FM4-64. Both ligands were independently tested against the control and found to be significantly higher (Student’s t-test, P < 0.005). Number of cells: control (n = 20), Wnt3a (n = 19) Wnt5a (n = 18). (**C**) Representative whole-cell capacitance recordings of control and Wnt-treated HEK293 cells. Arrow marks the time point of addition of the Wnt protein. Ga and Cm: imaginary and real part of admittance, corresponding to changes in membrane conductance (Ga lower trace) and capacitance (Cm upper trace). (**D**) Quantification of the effect of the Wnt ligands in whole-cell capacitance measurements. Number of measured cells: control (n = 9), Wnt3a (n = 9) Wnt5a (n = 10).

We used recombinant human canonical Wnt3a and noncanonical Wnt5a protein to analyse their endocytic uptake into HEK293 cells as a model system. These cells contain the Wnt signalling machinery endogenously and exhibit a robust Wnt response^18,32,33^. In a first assay, HEK293 cells were incubated with an endocytosis marker, the styryl dye FM 4-64 (10 µM) and subsequently challenged with either recombinant Wnt3a or Wnt5a (5 ng/ml). The confocal data in Fig. 1A show that unstimulated HEK293 cells exhibited a constant rate of endocytosis. Addition of Wnt3a or Wnt5a to the bath strongly increased endocytosis. While the response to Wnt3a was immediate, Wnt5a-stimulated endocytosis seemed to occur only after a lag of five minutes. After 30 minutes, it even exceeded the amount of endocytosis in Wnt3a-treated cells (Fig. 1B).

The stimulated uptake of FM 4-64 dye suggests that Wnt ligands induced endocytosis as an early step in the signal transduction cascade. Motivated by these findings, we next analysed the rapid effect of Wnt ligands on endocytosis by whole-cell patch-clamp capacitance measurements. Figure 1C shows a representative recording of an unstimulated HEK293 cell. These cells typically exhibited a stable capacitance of 4–15 pF. This corresponds to a membrane area of 500–1875 µm2 (diameter of 12–24 µm), an area typical for HEK293 cells. The recordings show that the Cm value remains mostly constant over the time of recording under control conditions. This implies that endocytosis (as visualised by the uptake of FM 4-64 dye in Fig. 1A) was balanced by exocytosis and thus a constant cell surface was maintained. The representative recordings in Fig. 1C show that exposure of the cells to Wnt3a and Wnt5a (5 ng/ml) in the bath solution caused an immediate and continuous decrease in the Cm value, indicating that both Wnt ligands stimulated endocytosis. In all tested cells, the Cm value dropped within one minute of stimulation by about 0.5 pF (±0.29 pF). Considering a specific membrane capacitance of 0.8 µF/cm^2^, this translates into a decrease in membrane area by 62 µm^2^ (±36 µm^2^) (diameter 4.5 µm ±3.4 µm). Figure 1D summarises the mean time course of ligand-stimulated endocytosis over ten minutes. Both ligands triggered a decrease in capacitance significantly below that of the background signal in control cells. The data further show that the ligand-stimulated increase in endocytosis approached saturation already about three minutes after stimulation. Furthermore, Wnt3a induced a maximal drop in the Cm values (2.2 pF ± 0.63 pF), thus exceeding the decrease induced by Wnt5a (1.6 pF ± 0.76 pF).

### Characterisation of steady state exo- and endocytosis in HEK293 cells

**Figure 2:**
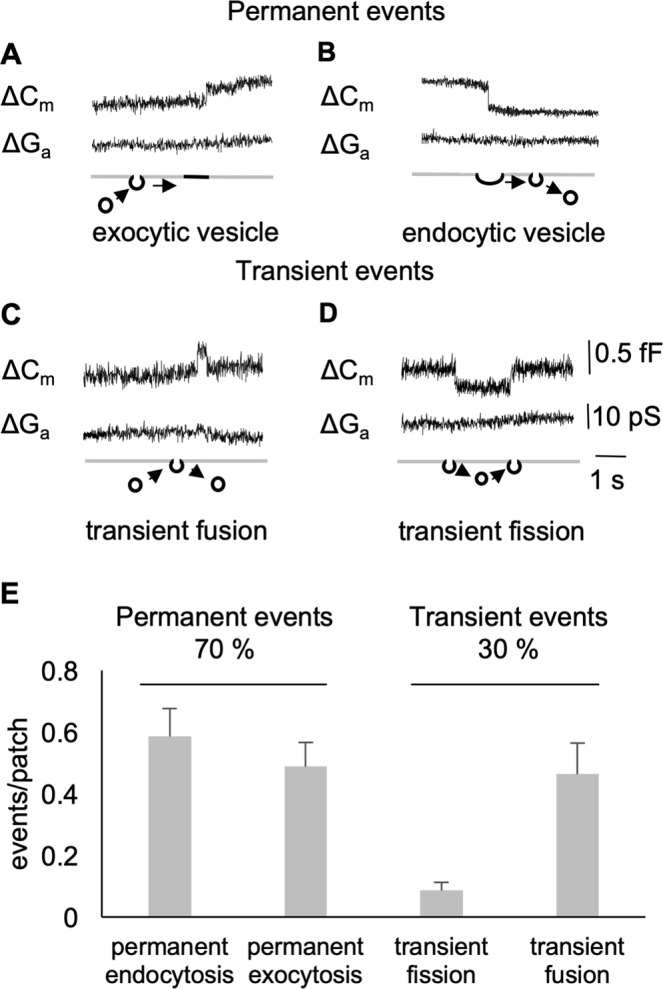
(**A**–**D**) Single spontaneous events (i.e. exocytosis, endocytosis and transients) in control HEK293 cells (imaginary and real part of admittance, corresponding to changes in membrane conductance (ΔG lower trace) and capacitance (ΔC upper trace)). (**E**) Number of permanent and transient events per patch, number of measured cells (n = 82), Each patch was measured on a separate cell and the first 500 s were used for the evaluation recording time in total.

To explore Wnt-stimulated endocytosis at the level of individual vesicle fission, we performed cell-attached patch-clamp capacitance measurements. For these recordings, we used pipettes with a resistance of 4–8 MΩ that translates into a tip opening of ~1 µm in diameter. The area under the pipette corresponds to less than 1% of the total membrane area of the cells investigated. In recordings of unstimulated cells, we occasionally observed spontaneous up- and downward changes of Cm, which reflect exo- and endocytosis of single vesicles within the patch, respectively (Fig. 2A–D). Spontaneous activity was observed in 56% of all patches (46 of 82 cells). In over 15 h of total recording time (ca. 11 min per patch), we observed on average only 2 events per cell. This spontaneous endo- and exocytotic activity caused capacitance steps between 0.08 fF and 7.6 fF. Considering a specific membrane capacitance of 0.8 µF/cm^2^ and a spherical shape of the vesicles, these capacitance steps translate into vesicle diameters of 56 nm to 550 nm.

Spontaneous C_m_ steps could be separated into four different categories (Fig. 2A–D) according to their kinetics: permanent exocytosis (A), permanent endocytosis (B), transient fusion (C), and transient fission (D). Most of the events are from permanent exo- and endocytosis (70%). Only 30% of the exo- and endocytic events are transient (Fig. 2E). Among these transient events, so called “kiss-and-run” exocytosis is more frequent (24%) than transient endocytosis (6%).

### Characterisation of receptor-mediated endocytosis of Wnt-treated cells

**Figure 3:**
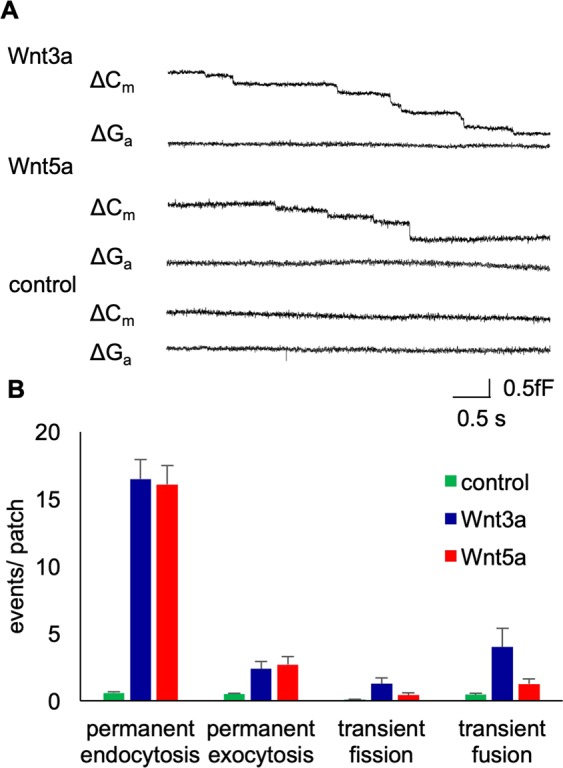
(**A**) Enhanced rate of endocytosis in Wnt-treated HEK293 cells. Representative capacitance recordings of Wnt3a and Wnt5a-treated HEK293 cells in the cell-attached mode showing successive downward steps corresponding to permanent endocytosis. Imaginary and real part of admittance, corresponding to changes in membrane conductance (ΔG upper trace) and capacitance (ΔC lower trace) for control, Wnt3a treated and Wnt5a treated HEK293 cells. (**B**) Number of permanent and transient events per patch in control (n = 82), Wnt3a (n = 37) and Wnt5a-treated (n = 41) HEK293 cells. Each patch was measured on a separate cell and the first 500 s were used for the evaluation.

**Figure 4:**
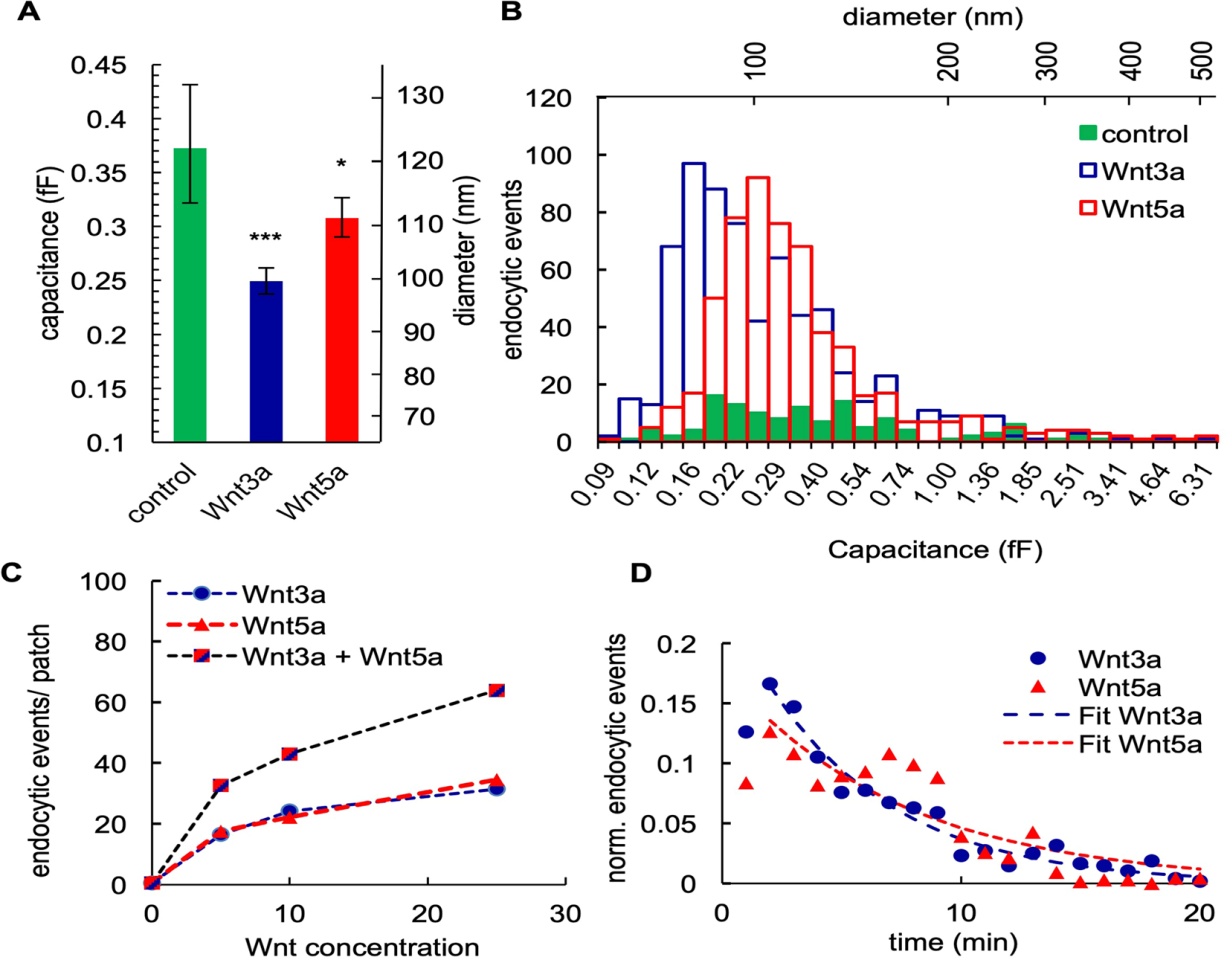
(**A**) Wnt-dependent size of endocytic vesicles. Left axis shows the geometrical mean (fF) and right axis the corresponding diameter (nm) of control, Wnt3a- and Wnt5a-treated patches. Endocytic vesicles stimulated by Wnt3a and Wnt5a are significantly smaller in vesicle size from the vesicles recorded in control cells (Error bars correspond to geometrical standard deviation for capacitance). Both ligands were independently tested against the control and against one another and found to be significantly different (Student’s t-test, ***P < 0.0005, **P < 0.005, *P < 0.05). Wnt3a and Wnt5a are also significantly different from each other**. (**B**) Distribution of different vesicle size intervals for both Wnt proteins and control condition. (**C**) Dose dependent increase of endocytic events per patch upon addition of different concentrations of Wnt3a and Wnt5a and simultaneous application of Wnt3a and Wnt5a. Each patch was measured on a separate cell and the first 500 s were used for the evaluation. Number of measurements: control (0 ng/ml; n = 82), Wnt3a (5 ng/ml; n = 37), Wnt5a (5 ng/ml; n = 41), Wnt3a (10 ng/ml; n = 6), Wnt3a (25 ng/ml; n = 3), Wnt5a (10 ng/ml; n = 8), Wnt5a (25 ng/ml; n = 5), Wnt3a + Wnt5a (2.5 ng/ml each; n = 19), Wnt3a + Wnt5a (5 ng/ml each; n = 4) and Wnt3a + Wnt5a (12.5 ng/ml each; n = 3). Under control conditions, 46 out of 82 measurements showed endocytotic events. In recordings with Wnt3a or Wnt5a in the pipette, we could detect endocytotic events in every measurement. (**D**) Temporal resolution of permanent endocytic events over 20 minutes and an exponential fit with tau 5.4 for Wnt3a and tau 7.4 for Wnt5a.

The presence of Wnt proteins in the extracellular solution (here 5 ng/ml in the pipette solution) caused a dramatic stimulation of endocytic activity in the membrane patches (Fig. 3A). After sealing of the micropipette with the plasma membrane, we monitored a robust and strong increase in permanent endocytic over exocytic activity (Fig. 3A–C). The exemplary traces in Fig. 3A, which were recorded immediately after sealing, showed frequent downward steps in the Cm signal. They reflect single endocytic events, which were only recorded with Wnt ligand present in the pipette. Compared to the control cells, the number of permanent endocytic events increased 20-fold for Wnt3a and 19-fold for Wnt5a within the timeframe of 10 min (Fig. 3B). Further control measurements confirmed that this stimulation was indeed triggered only by active Wnt ligands because the inactive Wnt ligands failed to stimulate endocytosis after boiling for 60 minutes. Figure 4D shows the temporal dynamics of Wnt-induced endocytosis. The number of endocytic events was high immediately after sealing of the pipette to the membrane and decreased exponentially thereafter. Fit of the data to an exponential function showed that Wnt3a-induced endocytosis decayed more rapidly (tau of 5.4 min) than Wnt5a-induced endocytosis (tau of 7.4 min). The time course of this stimulation of single endocytotic events occurred in the same order of magnitude as the was similar to Wnt-induced decrease in the capacitance in whole cell recordings (Fig. 1D). This implies that the rapid Wnt-induced increase in endocytotic activity is responsible for the observed decrease in the Cm value of the cells.

### Different kinetics of receptor mediated endocytosis in Wnt3 and Wnt5 treated cells

Next, we analysed the size distribution of endocytic vesicles, which are triggered by either Wnt3a or by Wnt5a ligands. We found that after stimulation with Wnt3a, endocytic vesicles had a geometrical mean of 0.25 fF +0.012/−0.011 fF (99 nm). For Wnt5a, we observed permanent endocytic vesicles with a geometrical mean of 0.31 fF +0.018/−0.017 fF (111 nm) (Fig. 4A). Both diameters were significantly smaller than those of vesicles from permanent endocytosis measured in unstimulated control HEK293 cells with 0.37 fF +0.063/−0.058 fF (122 nm). This suggests that Wnt ligands were endocytosed by vesicles that differed from the endocytic background activity in HEK293 cells.

To explore whether Wnt3a and Wnt5a are taken up by the same type of vesicle, we compared the distribution of the vesicle sizes induced by both ligands. Clathrin-coated vesicles tend to be larger (70–200 nm)^34^ than vesicles in clathrin-independent endocytosis (50–100 nm)^35–37^, thus differences in vesicle size may suggest different uptake mechanisms. Figure 4B shows the size distribution of Wnt3a and Wnt5a-induced endocytotic vesicles; the data indicate that Wnt3a-induced vesicles are smaller in size than Wnt5a-triggered vesicles.

**Figure 5:**
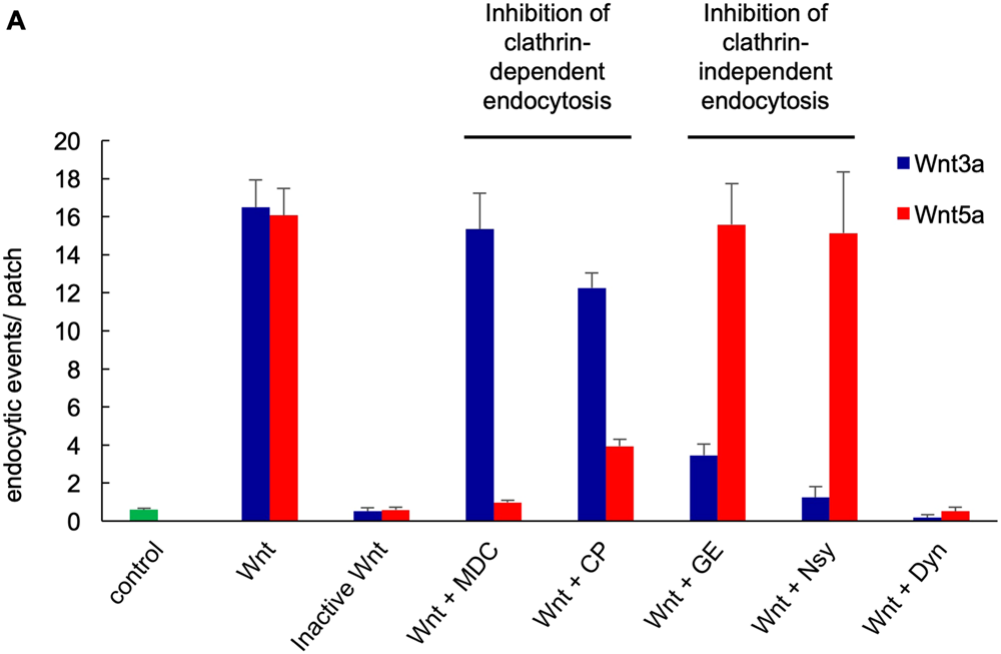
(**A**) Inhibition of Wnt-induced endocytosis by inhibitors of clathrin-dependent (Monodansylcadaverin MDC and Chlorprozamine CP) and clathrin-independent endocytosis (Genistein GE and Nystatin Nys). Number of permanent endocytic events per patch in control, Wnt3a and Wnt5a-treated HEK293 cells with the different inhibitors. Wnt3a cannot be blocked by MDC and CP, inhibitors of clathrin-dependent endocytosis, but is blocked by GE and Nys, inhibitors of clathrin-independent endocytosis. Wnt5a-induced endocytosis can only be blocked by MDC and CP, inhibitors of clathrin-dependent endocytosis. Dynasore (Dyn) blocked Wnt3a and Wnt5a endocytosis. Each patch was measured on a separate cell and the first 500 s were used for the evaluation. Measurement in total were: control (n = 82, Wnt3a (5 ng/ml; n = 37), Wnt5a (5 ng/ml; n = 41), Inactive Wnt3a (5 ng/ml; n = 8), Inactive Wnt5a (5 ng/ml; n = 7), Wnt3a + MDC (n = 9), Wnt5a + MDC (n = 9), Wnt3a + CP (n = 8), Wnt5a + CP (n = 14), Wnt3a + GE (n = 11), Wnt5a + GE (n = 12), Wnt3a + Nys (n = 13), Wnt5a + Nys (n = 11), Wnt3a + Dyn (n = 6), Wnt5a + Dyn (n = 6).

Because it is well established that cells can simultaneously use different endocytic pathways, we tested to what extent independent mechanisms are acting in the endocytosis of Wnt3a and Wnt5a. To discriminate between different pathways (i.e. clathrin, caveolin- or flotillin-dependent endocytosis), we analysed Wnt 3a and Wnt5a signalling under the influence of specific artificial inhibitors of clathrin-dependent and clathrin-independent endocytosis. The data in Fig. 5A show that Wnt3a and Wnt5a-triggered endocytosis could indeed be pharmacologically separated. Wnt3a-induced endocytosis was only slightly lowered by inhibitors of clathrin-mediated endocytosis. Monodansylcadaverin and Chlorprozamine reduced endocytosis of the Wnt3a ligand by 7% and 25%. However, Wnt3a-induced endocytosis was strongly reduced by inhibitors that block clathrin-independent endocytosis, e.g. Genistein and Nystatin. Both Genistein and Nystatin reduced Wnt3a-triggered endocytosis to 20.9% or 7.5% of the initial activity, respectively. An inverse response was found for the endocytosis of the Wnt5a protein. The endocytic activity triggered by this ligand was largely insensitive to inhibitors of clathrin-independent endocytosis; Genistein and Nystatin reduced Wnt5a-triggered endocytosis by 3% and 6%, respectively. In contrast, inhibitors of clathrin-dependent endocytosis caused a severe inhibition of Wnt5a-triggered endocytosis reducing it by 96% (Monodansylcadaverin), or 94% (Chlorprozamine), respectively. The results of these experiments corroborate the hypothesis that Wnt3a and Wnt5a are taken up into cells by two distinct and independent mechanisms. While Wnt5a presumably enters the cell via clathrin-coated vesicles, Wnt3a takes an independent route and uses a clathrin-independent endocytic pathway.

To further investigate the clathrin-dependence of the Wnt5a endocytosis, we used Dynasore to inhibit the small GTPase dynamin that is involved in scission of clathrin-coated vesicles. As shown in Fig. 5A, the uptake of both Wnt5a and Wnt3a is almost completely blocked by Dynasore. Even though unspecific side effects have been reported for Dynasore^38,39^, these data imply that dynamin may be essential for both endocytic routes in Wnt signalling (Fig. 5A).

We then analysed the concentration dependency of endocytosis for both Wnt ligands. The data in Fig. 4C show that endocytosis stimulation was equally concentration-dependent for both ligands. Importantly, both Wnts exhibited an additive effect when added simultaneously. When both Wnt proteins were administered together via the pipette solution at total concentrations of 5 ng/ml (2.5 ng/ml each), 10 ng/ml (5 ng/ml each) and 25 ng/ml (12.5 ng/ml each), the stimulating effect of both ligands on endocytosis was approximately additive at all concentrations tested. The most conclusive interpretation of the results of these experiments suggests a system in which both Wnt ligands use different and parallel endocytic pathways.

## Discussion

This study shows direct *in vivo* evidence for receptor-mediated endocytosis in Wnt signalling. High-resolution capacitance measurements report an enhanced fission of single endocytic vesicles in real time that was triggered in a specific manner by two tested Wnt ligands. The frequency of permanent endocytosis events increased approx. 19-fold over the background activity upon addition of canonical Wnt3a and noncanonical Wnt5a proteins in untreated HEK293 cells. These results demonstrate that Wnt signalling induces a fast and substantial increase in permanent endocytotic activity. The fact that transient endocytotic activity was not affected to the same extent suggests that Wnt receptor-mediated endocytosis relies mostly on a permanent uptake of vesicles from the plasma membrane. A quantification of the size of the endocytic vesicles induced by the Wnt ligands shows that they were significantly smaller than endocytotic vesicles recorded under control conditions. This suggests that the Wnt ligand does not increase the fission frequency of vesicles that are endocytosed in a constitutive manner. The distinct size of vesicles that are endocytosed in the presence of the ligand rather suggests that a specific type of vesicle is formed by a highly-regulated receptor-mediated endocytosis.

With the use of pharmacological inhibitors, the Wnt-triggered mechanism of receptor-mediated endocytosis can be further dissected into two discrete routes with and without clathrin. The receptor-mediated endocytosis of Wnt3a could be mostly blocked with Genistein and Nystatin, which are both inhibitors of clathrin-independent endocytosis. This finding is in accordance with several studies that showed that canonical Wnt3a signal activation is clathrin-independent^18–20^. Inhibition of clathrin-mediated endocytosis by Monodansylcadaverin and Chlorprozamine on the other hand leads to a strong reduction of receptor-dependent endocytosis of Wnt5a. Collectively, these data confirm the hypothesis of two parallel routes for endocytosis in the canonical and noncanonical Wnt pathways. The endocytosis of Wnt5a strongly depends on clathrin-mediated endocytosis, whereas Wnt3a is internalised to a great extent via clathrin-independent mechanisms. Since both pathways are blocked by the inhibitor Dynasore, the data further suggest that the small GTPase dynamin is involved in the fission of both clathrin-dependent and independent vesicles in this process. Under the assumption that the inhibitory effect of Dynasore is based on an interaction with dynamin, there is reason to assume that the Wnt3a ligand may enter cells via caveolin-dependent endocytosis. It is well established that this pathway equally relies on dynamin^40^. However, our mechanistic conclusions are based on pharmacological inhibitors and are thus limited by potential off-target effects^39,41^. To minimise a wrong interpretation of such unspecific side inhibitor effects, we used more than one pharmacological inhibitor per endocytosis pathway.

It has been assumed that the blocking of one pathway of receptor-mediated endocytosis may promote entry through another pathway, which is not important under physiological conditions^42^. However, our data contradict this hypothesis; they show that Wnt5a is not endocytosed by any alternative endocytic pathway when clathrin-mediated endocytosis is inhibited. This finding corroborates that Wnt5a enters the cell exclusively via clathrin-coated vesicles. For Wnt3a, the situation is more complex. Blocking of clathrin-dependent endocytosis with Chlorprozamine caused a small 25% reduction in endocytosis. This suggests that Wnt3a can enter cells to a small extent also via clathrin-mediated endocytosis. Several studies indeed suggest a role of such clathrin-mediated endocytosis also in canonical Wnt3a signalling. Yamamoto *et al*.^13^ showed that clathrin-mediated endocytosis is important for Wnt3a signal inhibition by sequestration of its receptor LRP5/6 from the plasma membrane in response to the Wnt antagonist Dkk1.

The concept of two parallel and mutually independent pathways for endocytosis of canonical and noncanonical Wnt signalling is further supported by experiments in which both ligands were administered together. Each of the Wnt proteins by itself induces endocytosis in a concentration-dependent manner. The application of both Wnt proteins together shows an additive effect in the sense that the total frequency of endocytosis exceeds the sum of the frequency, which is achieved by each individual ligand. The evidence for parallel and mutually independent pathways is further supported by our finding that specific inhibitors of one pathway do not interfere with the other endocytotic pathway. Both Wnt proteins do not depend on the same pathway for endocytosis because the number of endocytic vesicles is not increasing linearly with the ligand concentration. Instead, the frequency of endocytosis reaches a saturation level, which can only be overcome by adding both Wnt ligands together. This behaviour would not be expected if both Wnt ligands were preferentially interacting or competing for the same pathway. Our finding of two independent endocytotic pathways is different from previous hypotheses postulating that the suppression of Wnt5a by Wnt3a can be explained by the competition of both ligands for the same Frizzled 2 receptor at the plasma membrane^43^.

Receptor-mediated endocytosis, which is triggered by both Wnt ligands, is a very fast process. Within the limits of the temporal resolution of capacitance measurements, the data show that ligand-triggered vesicle fission occurs immediately upon receptor binding and ceases again after a few minutes. This fast triggering of endocytosis suggests an active role of endocytosis in both canonical and noncanonical Wnt signalling. The whole signalling complex, as well as the receptor-endocytosis machinery, must be present in or in close proximity to the plasma membrane. This assumption is consistent with data from Yang *et al*.^44^ who showed that endogenous Axin, a crucial component of the early Wnt pathway, is already present at the plasma membrane in puncta in *Drosophila* without stimulation through the Wnt pathway.

In the case that receptor-mediated endocytosis determines the onset of the Wnt signalling cascade, it might be expected that downstream signalling events occur only after this initial trigger. The first step after binding of Wnt to its receptor is the activation of GSK3, which leads to the formation of the LRP6-signalosome, phosphorylation and recruitment of Axin to this complex and the ensuing stabilisation of ß-catenin in the cytosol followed by its translocation into the nucleus.

Recent studies of the time course of the intracellular canonical Wnt signalling cascade indeed indicate a dynamic that fits well with the dynamics of receptor-mediated endocytosis demonstrated in our measurements. For canonical Wnt3a signalling, Ding *et al*.^45^ showed a GSK3 activity within the first ten minutes after Wnt addition. The phosphorylation of Axin is detectable shortly after Wnt 3a treatment in HEK 293 cells and is diminished after 15 to 30 minutes^44,46^. This means that both events follow directly after the endocytic activity that is observed in our measurements.

This sequence of events highlights the fact that the fast and vesicle-specific endocytosis of Wnt ligands occurs as a first initial step guiding the intracellular Wnt signalling cascade towards canonical and noncanonical Wnt signalling.

## Conclusions

In this study, we show direct evidence for receptor-mediated endocytosis in Wnt signalling. High resolution membrane capacitance measurements are able to resolve the very fast kinetics of Wnt3a and Wnt5a endocytotic uptake in real time. By using specific inhibitors, we could distinguish between canonical and noncanonical pathways and demonstrate that the internalisation of Wnt5a depends exclusively on clathrin-mediated endocytosis, whereas Wnt3a uptake was mostly driven by clathrin-independent mechanisms. The crucial role of dynamin in both endocytic pathways could be shown by an almost complete block in endocytosis after treatment with the inhibitor Dynasore. Since receptor-mediated endocytosis also plays an important role in many other morphogen signalling pathways such as Notch, Hedgehog and TGFß-signalling, we expect many more applications for high resolution patch-clamp capacitance studies.

Taken together, these results underline the importance of receptor-mediated endocytosis in Wnt signalling and provide the basis for identifying new molecular players in the early differentiation of canonical and noncanonical Wnt signalling.

## Materials and Methods

### Electrophysiology

Patch pipettes with a tip resistance in the range of 4–8 MΩ were prepared daily from glass capillaries (Kimax 51, Kimax Products, Vineland, NJ, USA), which were coated with Sigmacote (Sigma-Aldrich, Munich). Pipettes were filled with bath solution. Single isolated cells were brought in contact with the pipette tip and a high resistance seal was achieved by suction. Suction on the patch pipette was ended once a seal was achieved.

Measurements were performed with a dual-phase lock-in patch-clamp amplifier (SWAM IIC, Celica, Ljubljana, Slovenia). Membrane patches were clamped at 0 mV on which a sine wave (root mean square 111 mV, sine wave frequency 1.6 kHz) was applied. The phase of the lock-in amplifier was adjusted to nullify changes in the real part (R_e_) of the admittance signal to a manually generated 100 fF calibration pulse. The output signals were low pass filtered (10 Hz, −3 db), acquired at 100 Hz by an A/D converter (NI-DAQ, National Instruments, Austin, USA) and stored on a personal computer. The signal in-phase reflects R_e_ and is equivalent to the patch conductance; the out-of-phase signal corresponds to the imaginary part (I_m_). If there is no reflection in the R_e_ trace, the Im trace is directly proportional to the C_m_ changes. For events with projections between R_e_ and I_m,_ the vesicle capacitance C_v_ and the pore conductance G_p_ were calculated according to Lollike and Lindau^47^:

$${{\rm{C}}}_{{\rm{v}}}=[({{{\rm{R}}}_{{\rm{e}}}}^{2}+{{{\rm{I}}}_{{\rm{m}}}}^{2})/{{\rm{I}}}_{{\rm{m}}}]/{\rm{\omega }}$$

where ω is the angular frequency (ω = 2πf) and

$${{\rm{G}}}_{{\rm{p}}}=({{{\rm{R}}}_{{\rm{e}}}}^{2}+{{{\rm{I}}}_{{\rm{m}}}}^{2})/{{\rm{R}}}_{{\rm{e}}}.$$

As the membrane capacitance is proportional to the membrane area, the diameter (d) of the vesicle can be determined from the vesicle capacitance according to the equation

$$\begin{array}{ll}{{\rm{C}}}_{{\rm{v}}}={{\rm{C}}}_{{\rm{spec}}}{}^{\ast }{\rm{A}} & {\rm{A}}={{\rm{\pi }}d}^{2}\end{array}$$

where C_spec_ (specific capacitance) is the capacitance per unit membrane area. It was set to 0.8 µF/cm^2^, a value that has previously been described for cellular membranes^29,48^. Events were detected manually using the cursor option in the software subroutine (CellAn, Celica, Slovenia) written for MATLAB (MathWorks Inc., Natik, MA, USA) with additional digital filtering as required. Values are presented as median or mean ± SEM. Data were stored as previously described^49^ in a MySQL Community Server 5.1.49 database (Oracle). Calculations were performed using the CAMMC web application and results were again stored in the database.

For whole-cell measurements, we only analysed data sets with a stable access conductance G_a_ over 100 nS during the entire measurement (45 min). Therefore, HEK293 cells were kept in an extracellular bath solution and the membrane patch of approximately 1 µm^2^ under the pipette stayed intact.

### Confocal imaging

HEK293 cells were imaged with a Leica TCS SP5 II spectral confocal microscope (Leica Microsystems). Images were acquired with an HCX PL APO CS 40 × 1.3 Oil UV object.

Cells were incubated in 10 µM FM 4-64 (Invitrogen, Darmstadt, Germany) for different time points and excited with the 488 nm line of a 25 mW Argon laser and fluorescence was detected at 630–700 nm. Images were analysed according to Bandmann *et al*.^50^ with ImageJ software (NIH). For analysis of the relative intracellular fluorescence the ratio of intracellular fluorescence (area below the outline of the plasma membrane) compared with the whole cell fluorescence was used. To discriminate the whole cell fluorescence from the intracellular fluorescence, the outline of the cell (indicated by the plasma membrane staining FM 4-64) was manually traced using Image J (NIH). Subsequently, this outline was reduced by the same value for every cell to ensure comparability. Values are presented mean ± SEM.

### Cell culture

HEK293T cells were grown in continuous cultures as previously described^51^. Recordings were made within 1–3 days after plating. Experiments were performed on cells incubated at 37 °C in 5% CO_2_ for 2 to 3 days. Cells were bathed in a bath solution containing the following: 20 mM KCl, 1.8 mM CaCl_2_, 1 mM MgCl_2_, and 5 mM HEPES at pH 7.4. Mannitol was used to adjust the osmolarity to 300 mOsmol/kg.

The purified recombinant Wnt3a and Wnt5a proteins were suspended with PBS, 0.1 mM EDTA, 0.5% CHAPS and 0.5 mg BSA (R&D Systems). They were used at 5, 10, 25 and 50 ng/ml.

### Inhibitors

Clathrin-dependent endocytosis was analysed by using Chlorprozamine hydrochloride (CP) and Monodansylcadaverin (MDC). Chlorprozamine hydrochloride (100 µM for 10 minutes) is a cationic amphiphilic drug that inhibits clathrin-mediated endocytosis by depleting clathrin and adapter protein 2 (AP2) from the plasma membrane^52–54^. Monodansylcadaverin (MDC) (1 mM for 30 minutes) blocks the enzyme transglutaminase 2, which is necessary for receptor crosslinking in the region of clathrin-coated pits. Both CP and MDC have been shown to selectively inhibit clathrin-mediated endocytosis^55^.

Caveolae-mediated endocytosis was inhibited by using Filipin III, Nystatin, Methyl-ß-cyclodextrin and Genistein. Filipin III and Methyl-ß-cyclodextrin (MßCD) deplete the membrane of cholesterol and therefore are widely used to inhibit caveolin-mediated endocytosis. Our experiments with Filipin III (3 µg/ml for 30 minutes) and MßCD (5 mM for 30 minutes) revealed that HEK293 cells were heavily affected by both treatments. After treatment with 5 mmol/l MßCD, we were not able to perform regular patch-clamp measurements because the formation of a GΩ-seal was not possible or the seal disrupted after a few minutes. The rate of transient events increased and permanent exocytosis was reduced to almost zero, which fits with the hypothesis that cholesterol also plays an important role in docking and fusing of exocytotic vesicles^56^. These inhibitors with strong side effects were excluded in this study. The Ionophor Nystatin (25 µg/ml for 30 minutes) disrupts the lipid raft containing caveolin. We also tested Genistein (200 µM for 30 minutes), which is a tyrosine kinase inhibitor that causes local disruption of the Actin network at the site of endocytosis and effectively inhibits caveolae-mediated endocytosis^53,57,58^. 200 µM Genistein did not show apparent toxic effects on HEK293 cells. Dynasore was used at 80 µM for 30 minutes and did not show toxic effects on HEK293 cells. Each inhibitor was added to the bath solution and HEK293 cells were incubated for 30 min before performing the measurements.
